# Educational and economic returns to cognitive ability in low- and middle-income countries: A systematic review

**DOI:** 10.1016/j.worlddev.2021.105668

**Published:** 2022-01

**Authors:** Sachiko Ozawa, Sarah K. Laing, Colleen R. Higgins, Tatenda T. Yemeke, Christine C. Park, Rebecca Carlson, Young Eun Ko, L. Beryl Guterman, Saad B. Omer

**Affiliations:** a Division of Practice Advancement and Clinical Education, UNC Eshelman School of Pharmacy, University of North Carolina, Chapel Hill, NC, USA; b Department of Maternal and Child Health, UNC Gillings School of Global Public Health, University of North Carolina, Chapel Hill, NC, USA; c Duke Global Health Institute, Duke University, Durham, NC, USA; d Health Sciences Library, University of North Carolina, Chapel Hill, NC, USA; e Hubert Department of Global Health, Rollins School of Public Health, Emory University, Atlanta, GA, USA; f Yale Institute of Global Health, Yale University, New Haven, CT, USA; g Department of Internal Medicine (Infectious Diseases), Yale School of Medicine, Yale University, New Haven, CT, USA; h Department of Epidemiology of Microbial Diseases, Yale School of Public Health, Yale University, New Haven, CT, USA

**Keywords:** Cognition, Cognitive ability, Returns, Educational attainment, Earnings

## Abstract

There is growing interest to use early cognitive ability to predict schooling and employment outcomes in low- and middle-income countries (LMICs). Rather than using educational attainment and school enrollment as predictors of future economic growth or of improving an individual’s earning potential, mounting evidence suggests that cognitive ability may be a better predictor. The relationship between cognitive ability, education, and employment are essential to predict future development in LMICs. We performed a systematic literature review and *meta*-analysis of the evidence regarding the relationship between cognitive ability and educational outcomes, and between cognitive ability and economic outcomes across LMICs. We searched peer-reviewed studies since 2000 that quantitatively measured these relationships. Based on an initial search of 3,766 records, we identified 14 studies, including 8 studies that examined the cognition-education link and 8 studies that assessed cognition-employment returns in LMICs. Identified studies showed that higher cognitive ability increased the probability of school enrollment, academic achievement, and educational attainment across LMICs. A *meta*-analysis of returns to wages from cognitive ability suggested that a standard deviation increase in cognitive test scores was associated with a 4.5% (95% CI 2.6%–9.6%) increase in wages. Investments into early cognitive development could play a critical role in improving educational and economic outcomes in LMICs. Further research should focus particularly in low-income countries with the least evidence, and examine the impact on education and economic outcomes by cognitive domains to provide more robust evidence for policy makers to take action.

## Highlights

• Cognition and early cognitive development are crucial to educational and economic outcomes, but the association is not widely studied in LMICs.
• Increasing cognition scores by one standard deviation is associated with a 4.5% (95% CI 2.6–9.6%) increase in wages across LMIC studies.
• Higher cognitive ability increased school enrollment, academic achievement, and educational attainment across LMICs.
• The benefits of early cognitive development should be considered within the context of later educational and economic returns.

## 1 Introduction

There is increased interest in predicting economic outcomes from early childhood abilities. Since Mincer’s seminal earnings function was published in 1974, health economists have utilized this model to estimate the returns to education around the world, offering variations of their own models to account for different factors in their analyses (Mincer, 1974). This research has mainly operated under the assumption that greater educational attainment leads to improved economic outcomes, measured by growth in Gross Domestic Product (GDP), increased earnings, and employment opportunities. As a result, over the past few decades, policy makers and stakeholders working in low- and middle-income countries (LMICs) have focused investments on interventions to improve school enrollment with the goal of spurring growth and economic stability (Becker, 1995, Glewwe, 2002, Hanushek, 1995, UNDP, 2016, World Bank, 2001). Compulsory primary school education and subsidy policies in many countries have led to higher enrollment and educational attainment. However, the success of these initiatives in improving economic returns is inconclusive. Initial studies showed that LMICs had substantially high rates of return to education (Jee-Peng & Emmanuel, 1986, Psacharopoulos, 1985, Psacharopoulos, 1994). Yet, some economists later began to suspect that these rates were inflated (Glewwe, 1996, Heckman and Vytlacil, 2001). Part of the reason for this inflation is thought to be related to biases in the indicator of total years of schooling, which was used to measure human capital. It has been argued that educational attainment and school enrollment may not be the most accurate predictor of future economic growth or of improving an individual’s earning potential and that focusing instead on cognitive ability may better predict human capital returns.

Cognitive ability is a general term that encompasses the many forms of intelligence. There are several distinct domains of cognitive abilities. Here we briefly describe the domains used in the literature that are included in this manuscript. General intelligence is mainly divided into fluid intelligence and crystallized intelligence, where the former depends on the person’s native ability to think, reason, and solve problems, while the latter is acquired through education and experience such as vocabulary, literacy, numeracy, and mathematical skills. Executive function and self-regulation skills include planning, paying attention, filtering distractions, memorizing instructions, multitasking and impulse control. Working memory or short-term memory is the ability to store and manage the information for a short period of time even if distracted. Further explanation on fluid and crystallized intelligence, executive function, and working memory can be found in these citations (Alloway and Alloway, 2015, Blair and Razza, 2007, Molfese et al., 2010, Schubert et al., 2019).

Cognitive ability measurement began as a way of identifying children likely to be academically successful and is applied in different realms such as in hiring and placing employees, in predicting academic achievement, and in psychology to understand the changes in cognition of patients impacted by mental illness or brain injury (Campbell et al., 2008, Marks, 2014). Cognitive processes cannot be directly observed, thus all indicators used to measure cognitive domains are inferences based on the theory of how cognition functions (Ewoldsen, 2017). Indicators include those for general intelligence such as the Wechsler Intelligence Scale, which combine tests for multiple domains (Grizzle, 2011, Weiss, Saklofske, Holdnack, & Prifitera, 2016). Fluid intelligence is measured through testing non-verbal reasoning and the ability to adapt to and solve novel problems. The Raven’s Progressive Matrices seeks to measure fluid intelligence using non-verbal investigation, thereby unlinking cognitive ability from language ability (Raven, Raven, and Court, 1998). Other cognitive ability indicators measure crystalized intelligence through academic achievement based on student academic performance or large national standardized tests. These can also be a proxy for measuring executive function (Jacob & Parkinson, 2015). Similarly, ability type tests which include general tests of literacy and numeracy have been used as measures of cognitive ability to estimate the impact of cognition on human capital (Hanushek and Woessmann, 2008, Hanushek and Woessmann, 2012, Hanushek et al., 2015).

As economies advance, higher cognitive skills may play a greater role in wage determination and employment options than total years of schooling completed (Glewwe, 1996). Concurrent advancements in the fields of psychology and educational research have led to the rise in research estimating the role of cognitive ability in returns to education (Liu et al., 2018, Rohde and Thompson, 2007). As a result, there is a growing body of evidence that suggests that cognitive ability may serve as a useful predictor of economic outcomes, both in terms of individual employment and subsequent earnings or national GDP growth (Glewwe, 1996, Hanushek and Woessmann, 2008). Furthermore, there are indications that these associations might be quite strong (Green and Craig Riddell, 2003, Hanushek, 2005). Older studies have found that an additional year of schooling may yield between 7 and 11% returns in earnings, and one standard deviation in cognitive ability test scores has been found to correlate with 10–15% in additional earnings in the United States (Hanushek and Woessmann, 2008, Lazear, 2003, Mulligan, 1999, Murnane et al., 2000). One hypothesized factor differentiating these predictors is that educational attainment does not account for education quality, which could potentially have a large impact on skills attainment (Hanushek & Woessmann, 2008). In addition, comparing the impact of educational attainment across countries becomes difficult because education quality varies greatly even within countries. Therefore, cognitive ability can be a more useful indicator for international comparisons.

Although there is robust evidence of the impact of cognitive ability on educational and economic returns from high-income countries (Heckman & Vytlacil, 2001), there is little summative evidence in LMICs (Barro & Lee, 1993). To our knowledge, no systematic reviews have been conducted on the educational and economic returns to cognitive ability in LMIC contexts. With the availability of more panel data from LMICs, and considering that future economic growth in LMICs may depend upon skills growth to adapt to ever increasing advancements in technology (Hanushek & Woessmann, 2008), there is a critical need for more current analyses across LMICs. Greater evaluation of the roles and interactions of different aspects of economic and human capital outcomes (such as health and education) would better inform policy makers on how and where to invest to generate the greatest impact. We sought to assess the existing literature on the educational and economic returns to cognitive ability in LMICs and offer considerations for future research.

## 2 Methods

### 2.1 Literature search

We searched eight major databases: ERIC (Education Resource Information Center), Education Full Text, PubMed, EconLit, PsycInfo, Scopus, Embase, and Sociological Abstracts. Searches were conducted in February 2019. We searched for studies that estimated the linkages between cognition, education, and employment. We included studies that reported quantitative estimates of the association between cognition and educational outcomes, or cognition and employment outcomes. Cognitive domains focused on fluid and crystallized intelligence, executive function, and working memory. We included studies that reported cognitive skills measured through standardized literacy or numeracy tests, given evidence from high-income countries that cognitive abilities were closely correlated with literacy and numeracy scores capturing crystallized intelligence (Marks, 2014). We included studies that reported educational outcomes such as academic achievement, educational attainment, or school enrollment. Studies that reported employment outcomes such as individual earnings or employment status were included. Studies conducted in LMICs were included based on the World Bank country categorization at the time of this study (World Bank, 2019a). Studies were excluded if they were published before 2000, used data collected prior to 2000, or did not have English full-text available. We excluded books, conference proceedings, working papers, and discussion papers.

We screened studies using Covidence software (Veritas Health, 2021). Each reference was screened by two reviewers, first by title and abstract and then by full text (SL, CH, TY, CP, RC, YK). Conflicts were resolved either by a third reviewer or through discussion between the two reviewers with conflicting results. Studies were assessed based on how well they captured cognitive domains of interest and quantitative linkages to educational and economic outcomes. Studies were excluded if they focused exclusively on high achievers or low achievers, or if they focused on specific cognitive disorders or special populations, where the results would not be generalizable to the general population. This study is reported using the Preferred Reporting Items for Systematic Reviews and Meta-analyses (PRISMA) reporting guidelines. A description of the full search strategy, including inclusion and exclusion criteria and search terms, is provided in Appendix A.

### 2.2 Data abstraction

For each study, we abstracted data on general study characteristics (author, title, country, year published), study data collection date, type of data (primary study vs. use of secondary panel data), and type of statistical analyses employed. We categorized countries by World Bank income status (low-income, lower-middle-income, and upper-middle-income) and noted the current GDP per capita for each study country (World Bank, 2019b). We extracted information on which cognitive domain was studied, and specific cognitive assessment tools (or elements of the tools) that were used to measure cognitive ability or skills. We categorized studies by outcome: studies of educational outcomes were grouped by years of schooling completed, probability of school enrollment, and academic achievement. Economic outcome studies were grouped by those examining wages or employment as outcomes. We also recorded the estimated effect size reported or the coefficient of the regression analysis if no other results were provided. For studies that reported wage or employment returns from multiple models with and without an education covariate, we recorded results which controlled for education. We noted whether studies reported effect sizes for one standard deviation increase in cognitive ability, general correlations or other effect estimates, alongside statistical significance. If the effect size for one standard deviation was not reported but could be calculated from the study, we multiplied the standard deviation by the regression coefficient to estimate comparable values.

### 2.3 Meta-analysis

We conducted a *meta*-analysis among a subset of studies that examined the economic returns to cognition based on a change in natural log of wages by one standard deviation change in cognitive ability test scores. This subset was chosen for the *meta*-analysis because multiple studies examined the returns to cognition on log-wages, whereas the returns to cognition on other outcomes were not reported in comparable metrics. Studies that met these criteria underwent a quality assessment modeled after the GRADE approach (Schünemann and Santesso, 2019, Schünemann et al., 2013). A quality scale was constructed and applied to each study to assess confidence in the data and results reported. Two reviewers (SL, CH) independently evaluated each study, then the average of the two scores were taken. Studies with quality scores below 2 on a scale of 0 to 5 were not included in the *meta*-analysis. Publication bias of studies included in the *meta*-analysis was investigated using a funnel plot of one standard deviation change in cognitive test score and the sample size of each study included in the *meta*-analysis (see Appendix). Average effect sizes of included studies were weighted by individual quality scores in the overall estimate to represent the return to wages for one standard deviation increase in cognitive test scores.

## 3 Results

### 3.1 Literature search

**Fig. 1:**
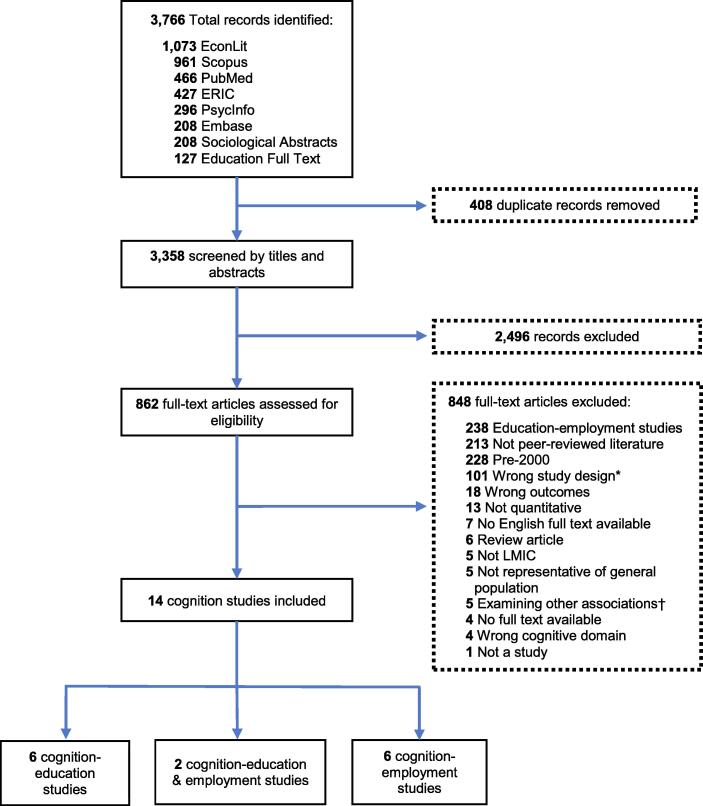
PRISMA flow diagram. ERIC = Education Resource Information Center; LMIC = Low- and middle-income country. *Wrong study design category included studies that did not meet the inclusion criteria due to how cognition, education, or employment were defined, evaluated, or modeled. † Examining other associations category included studies where the dependent variable was cognition and independent variable was either employment or education, or studies where the dependent variable was education and the independent variable was employment.

Our searches yielded a total of 3,766 citations across the eight databases, with over a quarter each (26%–28%) from EconLit (1073 records) and Scopus (961 records), followed by over 10% each from PubMed (466 records), and ERIC (427 records). Few records were obtained from PsycInfo (296 records), Embase (208 records), Sociological Abstract (208 records) and Education Full Text (127 records). After removing duplicates, we identified 3,358 articles, which were screened based on titles and abstracts. We reviewed the full-texts of 862 studies, of which 14 cognition studies met all of our inclusion criteria (Akresh et al., 2012, Aslam et al., 2012, Campos-Vazquez, 2018, Chua, 2017, Fink et al., 2015, Glewwe et al., 2017, Glick and Sahn, 2010, Haile et al., 2016, Hannum et al., 2013, Millones et al., 2011, Sun et al., 2018, Sun, 2019, Tan and Thamarapani, 2018, Yu, Wang, Shen, Shi, & Li, 2017). Two of the included studies measured both educational and economic outcomes (Glewwe et al., 2017, Tan and Thamarapani, 2018). Fig. 1 summarizes our literature search process.

#### 3.1.1 Cognitive ability and educational outcomes

We found eight studies estimating the relationship between cognitive ability and educational outcomes in LMICs (Akresh et al., 2012, Fink et al., 2015, Glewwe et al., 2017, Glick and Sahn, 2010, Haile et al., 2016, Millones et al., 2011, Sun et al., 2018, Tan and Thamarapani, 2018). Fig. 2a describes the characteristics of these studies, where three studies measured school enrollment (Akresh et al., 2012, Fink et al., 2015, Glewwe et al., 2017), another three measured the effect of cognition on academic achievement (Haile et al., 2016, Millones et al., 2011, Sun et al., 2018), and three studies measured the impact of cognition on educational attainment (Glewwe et al., 2017, Glick and Sahn, 2010, Tan and Thamarapani, 2018). The most common cognitive domains examined were fluid intelligence (n = 4) (Akresh et al., 2012, Fink et al., 2015, Haile et al., 2016, Millones et al., 2011), executive function (n = 3) (Fink et al., 2015, Sun et al., 2018, Tan and Thamarapani, 2018), crystallized intelligence measured by literacy and numeracy (n = 3) (Fink et al., 2015, Glewwe et al., 2017, Glick and Sahn, 2010), and working memory (n = 2) (Akresh et al., 2012, Sun et al., 2018). Three studies each were conducted in low-income (n = 3, Burkina Faso, Ethiopia and Senegal) and lower-middle income countries (n = 3 studies in multiple countries; Cambodia, Ghana, Mongolia, Vanuatu, Zambia), with two studies in upper-middle income countries (n = 2; China, Peru). Included studies were a mix of primary studies (n = 4) and those that used panel survey data (n = 4).

**Fig. 2a:**
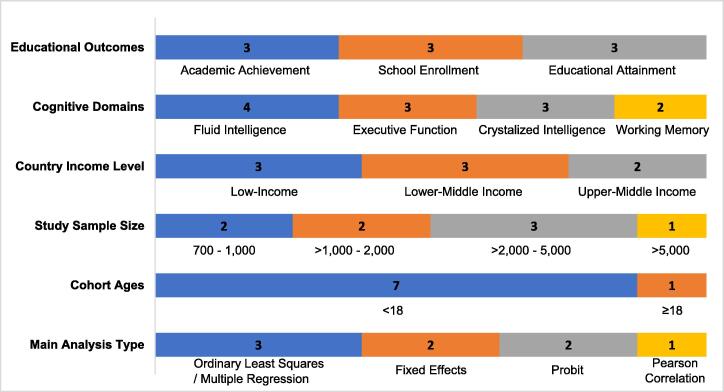
Overall study characteristics examining cognitive ability and educational outcomes.

Table 1 presents the eight studies that estimated the educational returns to cognitive ability in LMICs. All studies (n = 8) showed a positive correlation or a statistically significant predictive effect of cognitive ability on educational outcomes. Among the three studies that assessed academic achievement, one study in Ethiopia reported a positive correlation between fluid intelligence, measured through Raven's Progressive Matrices, and academic achievement (Haile et al., 2016). A second study in Peru showed that an increase in fluid intelligence, measured similarly, corresponded with an increase in academic achievement of 1.78–6.16 points on spelling, arithmetic, and reading tests (Millones et al., 2011). Another study across three countries (Mongolia, Cambodia, and Vanuatu) found that higher executive function led to higher academic achievement through improved scores in mathematics, literacy, and language, but the study did not provide the standard deviations of the cognitive test scores (Sun et al., 2018).

**Table 1:** Educational returns to cognitive ability in LMICs.

Author and year	LMIC countries studied	World Bank Income Level	GDP per capita (2017 USD)	Cognitive Domains	Cognitive Assessment Tool (Used or Incorporated)	Data Source	Data Years	Sample Size	Cohort Ages	Estimated Effect Size	Unit of measure	Impact of cognition on employment
Akresh et al., 2012	Burkina Faso	L	$642	Fluid intelligence, working memory	Raven's Colored Progressive Matrices, Wechsler Intelligence Scales - Digit Span	Burkina Faso Social Protection Evaluation Survey	2008, 2009	4,641	5–15 years, grade 2 or lower, grade 1 or lower	0.164–0.22*	Probability of school enrollment	One SD in cognitive ability increases the probability of a child being enrolled in school by 16.4%–22% controlling for age, gender, household & age fixed effects.
Fink et al., 2015	Zambia	LM	$1,513	Crystallized intelligence (language skills), fluid intelligence, executive function	Local version of Peabody Picture Vocabulary Test, NEPSY block test, Tactile Patterns Reasoning, "Pencil Tap" test	Zambia Early Childhood Development Project	2009	2,711	5–7 years	0.02–0.13*	Probability of school enrollment	One SD increase in executive function increases the likelihood of early and on-time school enrollment by 2%–13% controlling for age, gender, household size, region, income.
Glewwe et al., 2017	China	UM	$8,827	Crystallized intelligence (literacy, numeracy, language)	Chinese Test, Math Test, Cognitive Skills Test, Literacy Test, Numeracy Test	Gansu Survey of Children and Families	2000, 2004, 2007–2009	2,000	Cognitive skills measured at 9–12 years; outcome measured at 17–21 years	0.029–0.08*	Probability of school enrollment	One SD increase in Chinese, math, or literacy increases the likelihood of still being enrolled in school five years later by 2.9%–8.0% controlling for age, experience, parent education, wealth, non-cognitive skills.
										0.20–0.30*	Years of schooling	One SD increase in Chinese and math skills predicted an increase in years of schooling by 0.24 and 0.20 years in 2000. In 2004, one standard deviation increase in literacy test predicted 0.30 years increase in years of schooling controlling for age, experience, parent education, wealth, non-cognitive skills.
Glick & Sahn, 2010	Senegal	L	$1,329	Crystallized intelligence (language and numeracy)	Standardized second-grade pretest and posttest score (French and math)	EBMS, PASEC	1997, 2003	834	Second grade, middle school ages (14–17)	0.22*	Probability of school attainment	One SD increase in second-grade pre-test score increases the probability of completing 6th grade by 0.22 controlling for gender, parent education, school quality variables, and rural.
Haile et al., 2016	Ethiopia	L	$768	Fluid intelligence	Kaufman Assessment Battery for Children (KABC-II), Raven's Colored Progressive Matrices	Study data	2013–2014	129,128	8–11 years	Math score:0.19–0.38; academic score:0.14–0.38	Correlation Coefficient, academic achievement	Positive correlation between cognitive function and mathematics and average academic score without control variables.
Millones et al., 2011	Peru	UM	$6,572	Fluid intelligence	Raven's Standard Progressive Matrices Test	Study data stratified by Local Educational Management Unit, school type, grade	2009	1,129	11–12 years	1.78–6.16*	Academic achievement score	One point increase in intelligence increases spelling achievement by 1.78 points, arithmetic achievement by 6.16 points, reading achievement by 4.46 points controlling for intelligence, school type, and gender.
Sun et al., 2018	Cambodia, Mongolia, Vanuatu	LM	$1,384	Executive function, Working memory	East Asia-Pacific Early Child Development Scales (EAP-ECDS)	EAP-ECDS	2013, 2014	3,331	36–71 months	0.37–0.62*	Academic achievement score regression coefficient	Executive function significantly predicts achievement in language, literacy, or mathematics in the three countries and plays a mediating role in the SES-- academic achievement pathwaycontrolling for age, gender, and rural location.
Tan & Thamarapani, 2018	Ghana	LM	$2,046	Executive function	“Simple” and “Advanced” tests for executive function	Ghana Education Impact Evaluation Survey	2003	738	25 years and older	0.78–1.011*	Years of schooling	High sustained attention may predict an increase in length of schooling by 8–12 months controlling for age, gender, locality, family size, IQ, height, BMI, parent education, school quality, school reform, household characteristics and interactions with locality.

BMI – body mass index; EBSM – Senegal Household Education and Welfare Survey; GDP – gross domestic product; IQ –intelligence quotiet; L – low-income country; LM – lower-middle income country; LMIC – low- and middle-inocme countries; NEPSY - A Developmental NEuroPSYchological Assessment; PASEC – Program on the Analysis of Education Systems of the Conference of Francophone Ministers of Education; SD – standard deviation; SES – socio-economic status; UM – upper-middle income country; USD – United States dollars.

*Results were statistically significant (p < 0.05).

Three studies examined the impact of cognitive ability on school enrollment. One standard deviation increase in executive function was associated with increased probability of school enrollment by 2%-13% in Zambia (Fink et al., 2015). Another study on fluid intelligence using Raven’s Progressive Matrices showed returns in school enrollment by 16%–24% in Burkina Faso (Akresh et al., 2012). A study in China found that a standard deviation increase in literacy score increased the probability of still being enrolled in school five years later by 2.9%–8.0% (Glewwe et al., 2017). This was the only study to associate cognitive ability at a younger age with later academic and economic outcomes.

Impact of cognition on educational attainment was assessed in three studies. One study found that a one standard deviation increase in Chinese language and math skills predicted an increase of 0.20–0.30 additional years of schooling (Glewwe et al., 2017). In Senegal, one standard deviation increase in second grade pre-test scores (primarily testing in math and French) was found to increase the probability of completing sixth grade by about 22% (Glick & Sahn, 2010). Finally, one standard deviation increase in executive function was associated with increased years of schooling by 8–12 months in Ghana (Tan & Thamarapani, 2018).

#### 3.1.2 Cognitive ability and economic outcomes

We found eight studies assessing the relationship between cognitive ability and economic outcomes (Aslam et al., 2012, Campos-Vazquez, 2018, Chua, 2017, Glewwe et al., 2017, Hannum et al., 2013, Sun, 2019, Tan and Thamarapani, 2018, Yu, Wang, Shen, Shi, & Li, 2017). Fig. 2b shows the characteristics of these studies, where seven studies estimated the impact of cognitive ability on earnings (Aslam et al., 2012, Campos-Vazquez, 2018, Chua, 2017, Glewwe et al., 2017, Hannum et al., 2013, Sun, 2019, Yu, Wang, Shen, Shi, & Li, 2017) and three studies estimated the association between cognitive ability and the probability of employment or employment type (Aslam et al., 2012, Hannum et al., 2013, Tan and Thamarapani, 2018). Studies reported data from eight lower-middle income countries – Bolivia, Georgia, Ghana, Kenya, Pakistan, Ukraine, Vanautu, and Vietnam (Aslam et al., 2012, Chua, 2017, Tan and Thamarapani, 2018) and three upper-middle income countries – Armenia, China, Mexico (Campos-Vazquez, 2018, Chua, 2017, Glewwe et al., 2017, Hannum et al., 2013; Sun, 2019). Many of the studies were conducted in China (n = 4) and in Ghana (n = 2). Most studies examining economic returns to cognitive ability captured crystallized intelligence measured by literacy or numeracy (n = 6) (Aslam et al., 2012, Chua, 2017, Glewwe et al., 2017, Hannum et al., 2013, Yu, Wang, Shen, Shi, & Li, 2017, Sun, 2019). Three studies also examined fluid intelligence (Aslam et al., 2012, Campos-Vazquez, 2018, Yu, Wang, Shen, Shi, & Li, 2017), and one study each examined executive function (Tan & Thamarapani, 2018), and working memory (Campos-Vazquez, 2018).

**Fig. 2b:**
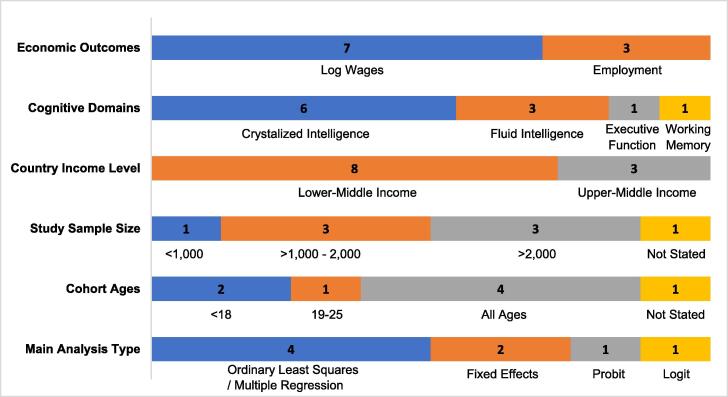
Overall study characteristics examining cognitive ability and economic outcomes.

Table 2 presents the economic returns to cognitive ability across LMICs. Four studies found a statistically significant positive association between cognitive ability and wage returns (Chua, 2017, Hannum et al., 2013, Sun, 2019, Yu, Wang, Shen, Shi, & Li, 2017). Three of these studies were conducted in China (Hannum et al., 2013, Sun, 2019, Yu, Wang, Shen, Shi, & Li, 2017), with one study finding that one standard deviation increase in crystallized intelligence measured by exam ability was associated with a wage increase of 6.7% (Sun, 2019). Another study found that one standard deviation increase in crystallized intelligence measured by literacy increased wages by 4.8%–5.6% (Hannum et al., 2013). One study which looked across eight different LMICs found that one standard deviation increase in cognitive ability, measured by literacy, increased wages by an average of 4.8% when controlling for schooling (Chua, 2017). Three out of seven studies on wage returns to cognitive ability showed an effect of cognitive ability on individual earnings that was initially statistically significant, but not after controlling for schooling (Aslam et al., 2012, Campos-Vazquez, 2018, Glewwe et al., 2017). A study in Mexico found one standard deviation increase in fluid intelligence to increase average monthly earnings by 4.7% when controlling for educational attainment, though the result was not statistically significant (Campos-Vazquez, 2018).

**Table 2:** Economic returns to cognitive ability in LMICs.

Author and Year	LMIC countries studied	World Bank Income Level	GDP per capita (2017 USD)	Cognitive Domains	Cognitive instruments used or Source of Cognitive Data	Data Source	Data Years	Sample Size	Cohort Ages	Estimated Effect Size	Unit of Measure	Impact of cognition on employment
Aslam et al., 2012	Pakistan	LM	$1,548	Fluid intelligence, crystallized intelligence (literacy, numeracy)	Raven's Progressive Matrices, literacy test, math test	Purpose-designed survey	2006–2007	1,194	15–60 years	(-)0.0094–0.0044	Wage employment	Literacy, math, and cognitive ability are not significantly associated with wage employment controlling for schooling, gender, work experience, and parent education.
										0.01–0.226	Monthly earnings	Literacy predicts earnings controlling for schooling, gender, work experience, and parent education.
Campos-Vazquez, 2018	Mexico	UM	$8,910	Fluid intelligence, working memory	Questions from Raven's Progressive Matrix, Wechsler Adult Intelligence Scale (WAIS-IV)	Mexican Social Mobility Survey	2015	2,616	adults	0.047	Monthly earnings	One SD increase in cognitive skill increases average monthly earnings by 4.7% controlling for years of schooling and non-cognitive skills.
Chua, 2017	Armenia, Bolivia, Colombia, Georgia, Ghana, Kenya, Ukraine, Vietnam	Blend UM/LM	$1,595 -$6,409	Crystallized intelligence (literacy)	Programme for the International Assessment of Adult Competencies (PIAAC), STEP Skills Measurement Program	PIAAC, STEP	2011–2013	NA	14–64 years	0.048*	Monthly earnings	One SD increase in literacy skills increases earnings by 4.8% controlling for gender, years of schooling, and work experience.
Glewwe et al., 2017^†^	China	UM	$8,827	Crystallized intelligence (literacy, numeracy)	Chinese test, math test, cognitive skills test, literacy test	Gansu Survey of Children and Families	2000, 2004, 2007–2009	2,000	Cognitive skills measured at 9–12 years; outcome measured at 17–21 years	0.013	Hourly earnings	One SD increase in cognitive skill increases earnings by 1.3% controlling for years of schooling, work experience, parent education, and non-cognitive skill variables.
Hannum et al., 2013^‡^	China	UM	$8,827	Crystallized intelligence (literacy, numeracy)	Chinese Adult Literacy Survey	China Urban Labor Survey	2002	Male: 859 Female: 802 Couples: 886	25–44 years	0.048–0.056*	Earnings	One SD increase in literacy is associated with increased earnings, controlling for years of schooling, work experience, training, communist party membership, and spouse characteristics. However, there are gender differences as less literate women tend to trade their income for spouses whereas more literate women are less likely to marry.
										0.142–0.286*	Probability of employment	One SD increase in literacy predicts the husband's employment status controlling for years of schooling, work experience, training, communist party membership, and spouse characteristics.
Sun, 2019	China	UM	$8,827	Crystallized intelligence (exam ability)	High School Entrance Exam score (HSEE/zhongkao), and National College Entrance Exam (NCEE/gakao)	Chinese Household Income Project (CHIP)	2002, 2007, 2013	4,404, 3,355, 4,097	16–60 years	0.067*	Hourly wages	One SD in exam ability increases wages by 6.7% controlling for age, gender, years of schooling, industry, province, public firm, and capital city. Exam score has a greater bearing on wages than schooling level or degree.
Tan & Thamarapani, 2018^§^	Ghana	LM	$2,046	Executive function	Ravens Progressive Matrices, mathematics, English reading	Ghana Education Impact Evaluation Survey	2003	738	>25 years	0.159–0.174*	Probability of employment	High levels of sustained attention are associated with 15.9%–17.4% increased probability of white-collar employment controlling for age, gender, locality, family size, IQ, height, BMI, parent education, school quality, school reform, household characteristics and interactions with locality.
Yu et al., 2017	China	UM	$8,827	Fluid intelligence, crystallized intelligence (literacy, numeracy)	PISA	Chinese Employer-Employee Survey	2015	5,364	adults	0.034–0.157*	Wages	Cognitive abilities are positively correlated with wages controlling for age, gender, marriage, education, and BMI.

BMI – body mass index; GDP – gross domestic product; IQ – intelligence quotient; L – low-income country; LM – lower-middle income country; LMIC – low- and middle-income countries; PISA - Programme for International Student Assessment; SD – standard deviation; UM – upper-middle income country; USD – United States dollars.

*Results were statistically significant (p < 0.05).

†Authors of the study state “there is no strong evidence that skills measured in childhood predict wages” but we report values from the tables.

‡Standard errors are wide, although results were reported as being statistically significant (p < 0.001).

§Results are extracted from Table 4A in the paper, which required there be no mistakes on the test for high sustained attention.

Of the three studies that examined employment outcomes (Aslam et al., 2012, Hannum et al., 2013, Tan and Thamarapani, 2018), one study in China found a positive association between a husband’s cognitive ability, measured by literacy score, and his employment status, but this was not found for the wife within paired relationships (Hannum et al., 2013). The second study found that those with higher executive function had a 15.9%-17.4% increased chance of obtaining white-collar work in Ghana (Tan & Thamarapani, 2018). The third study did not show a statistically significant association between fluid intelligence measured through the Raven's Progressive Matrices on wage employment status after controlling for schooling in Pakistan (Aslam et al., 2012).

The control variables included in each model can lend to different interpretations of the results. We report the coefficients from models that included the most common confounders, to make appropriate comparisons across studies. Most studies controlled for characteristics known to be influential in determining employment outcomes such as gender, age, and years of schooling. Some were able to control for family background characteristics such as wealth or parental education and one controlled for years of work experience (Campos-Vazquez, 2018, Chua, 2017, Glewwe et al., 2017, Sun, 2019, Tan and Thamarapani, 2018). Among studies that provided model results with and without covariates, inclusion of these covariates always led to a decrease in the coefficient on the cognitive ability variable. The most important covariate in examining the effect of cognition on employment outcomes was educational attainment, usually measured though years of schooling. One study found that including years of schooling as a covariate resulted in a decrease of the literacy score coefficient on log wages from 0.14 to 0.048 (67% decrease) across multiple countries (Chua, 2017). Similarly, the coefficient on working memory decreased from 0.14 to 0.047 (67% decrease) when years of schooling was included in a study conducted in Mexico (Campos-Vazquez, 2018).

### 3.2 Meta-Analysis

**Fig. 3:**
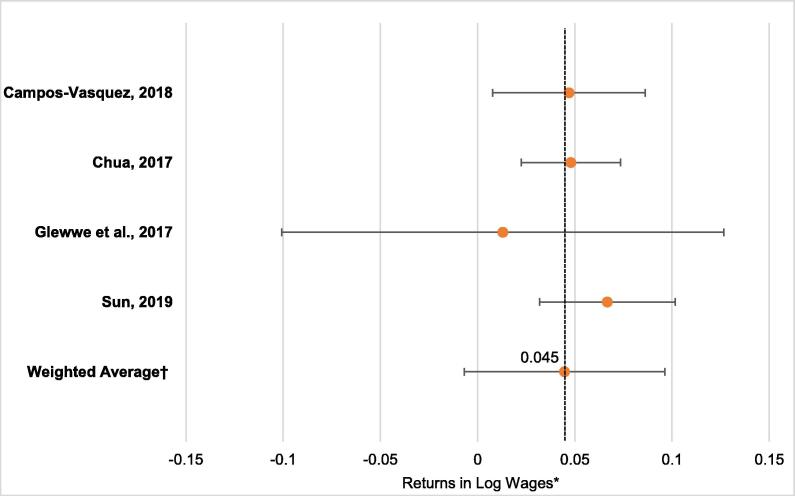
Forest Plot of Wage Returns to Cognition Test Scores*. *Point estimates reflect the returns in natural log of wages to one standard deviation increase in cognition test scores from each study. †The weighted average returns were weighted by quality scores.

Among five studies with results on wage returns to cognitive ability, four studies received high enough quality scores to be included in the *meta*-analysis (Campos-Vazquez, 2018, Chua, 2017, Glewwe et al., 2017, Sun, 2019). A forest plot of the return to natural log of wages for one standard deviation increase in cognitive test scores is presented in Fig. 3. After weighing by the quality of the studies, the average return was a 4.5% (95% CI 2.6%–9.6%) increase in wages for one standard deviation increase in cognitive ability. Based on the funnel plots and the limited number of studies, we cannot rule out the potential role of publication bias in our results.

## 4 Discussion

Our findings from the systematic review provide evidence in support of higher levels of cognitive ability predicting improved schooling and employment outcomes in LMICs. We found that higher cognitive ability may predict greater and longer school enrollment (Akresh et al., 2012, Fink et al., 2015, Glewwe et al., 2017), academic achievement through improved academic test scores (Haile et al., 2016, Millones et al., 2011, Sun et al., 2018), and educational attainment (Glewwe et al., 2017, Glick and Sahn, 2010, Tan and Thamarapani, 2018). Wage returns to cognitive ability in LMICs over the last 20 years from our *meta*-analysis across four studies saw a 4.5% (95% CI 2.6%−9.6%) increase in wages for one standard deviation increase in cognitive ability (Campos-Vazquez, 2018, Chua, 2017, Glewwe et al., 2017, Sun, 2019). Employment opportunities also increased with cognitive ability, including the probability for white-collar work (Tan & Thamarapani, 2018).

These findings suggest that cognitive ability could serve as an indicator of the success of education and economic policy interventions and can aid in the continued evaluation and prioritization of investments in LMICs (Hanushek, 1995). There is evidence that increased cognitive skills and abilities may matter more than schooling attainment in wage determination, economic opportunity, and economic growth (Hanushek & Woessmann, 2008). Investors should therefore consider early childhood development programs that improve cognitive ability (e.g. improving educational quality) over those focusing on traditional indicators, such as school enrollment rates or increased graduation rates in LMIC contexts. Subsidizing primary education and encouraging compulsory education for all are important policies to continue, as primary education is still critical for building foundational skills (United Nations Educational & Scientific, 2015). Furthermore, there are other external benefits to school enrollment apart from future earnings and employment. This includes improved health outcomes such as lowered fertility rates for girls, which can have a substantial subsequent impact on their socio-economic future, particularly in LMICs (Pradhan, 2015, United Nations Populations Fund, 2013, World Health Organization, 2012).

Since early childhood cognitive abilities could play a key role in the educational trajectories and later economic successes of individuals living in LMICs, investments should also be made in initiatives affecting *in utero* and early childhood development such as nutrition and vaccination interventions (Anekwe et al., 2015, DiGirolamo et al., 2020). Poor maternal health can cause infants to be born with low birth weights (Bird et al., 2017, Shakya et al., 2015, Yilgwan, Utoo, & Hyacinth, 2012), leading to poorer neonatal health, which can impact cognitive development (Figlio, Guryan, Karbownik, & Roth, 2014). In contrast, there is some evidence that maternal immunization against influenza can improve infant birth weights (Steinhoff et al., 2012), and may also improve cognitive function (Xia, Qi, Zou, Yang, & Yao, 2014). Another study on children born to mothers receiving tetanus immunization showed that children born to immunized mothers had better educational attainment in Bangladesh (Canning et al., 2011). Additionally, studies have found that childhood stunting, which is common across LMICs (Victora, de Onis, Hallal, Blossner, & Shrimpton, 2010), can reduce cognitive function and subsequent earnings later in life (Fink et al., 2016, Vogl, 2014). One study found that a one standard deviation increase in cognitive ability had a similar impact on improving health outcomes as two years of schooling (Christopher Auld & Sidhu, 2005).

Our findings are in line with an earlier review on economic returns to cognitive abilities across mainly LMICs (Ghana, Kenya, Morocco, Pakistan, South Africa, Tanzania) that reported returns from 11 cognitive ability studies, which ranged between 5% and 48% (Hanushek & Woessmann, 2008). Lower estimates (5%) were associations between cognition and household income (Jolliffe, 1998) and higher estimates of 34%-48% reflected the association between mathematics skills and wages, which came from a study estimating the effect of the Apartheid schooling system in South Africa (Moll, 1998). The rest of the economic returns to cognitive ability reported in this earlier review were found to be between 7% and 28%. All of the studies in this earlier review utilized data prior to 2000, and were therefore not included in our analysis. Our results fall within the lower end of the range of returns to cognition reported previously. A number of differences could contribute to our lower estimate, namely the difference in time period (articles primarily conducted in the 1990′s versus 2000 and later) and our explicit choice to include estimates from modeled results that controlled for schooling, unlike prior studies.

Given the limited number of studies found across LMICs, there is a need for additional research in the area of cognitive returns in LMIC contexts. This would provide more robust evidence for policy makers in decision-making, particularly when weighing investments in education expansion versus improving education quality (Hanushek, 1995). However, it may be difficult to disentangle the impact of cognition from educational attainment in these comparisons. Most studies (n = 8) relied at least part of their analysis on literacy, language, math, or other school subject exams testing crystalized intelligence, which may be confounded by schooling. In addition, including educational attainment measured by years of schooling in regression models always decreased the effect of cognition. This indicates that students with higher cognitive ability do better in formal education and tend to go on to pursue higher levels of schooling, which would also result in more earnings (Chua, 2017). More studies that utilize established tools to measure aspects of fluid intelligence, executive function, and working memory in LMICs could help to direct the discussion of school quality over quantity. Regional differences could also be explored with additional data. Research should focus on low-income countries in particular, that were markedly underrepresented in research on cognitive returns (3 of 14 studies). Such evidence would also make a greater case for other interventions that can maintain or enhance cognitive abilities, such as vaccination, by virtue of preventing diseases that could affect cognitive development (Anekwe et al., 2015, Bloom et al., 2011).

More consistent use of rigorous measurements in LMICs is also called for. Given that school quality varies significantly across countries, future studies need to address measuring standard scores of cognitive abilities that can account for cross-country variations. Research should adapt and validate suitable cognitive assessment tools in LMIC contexts, including those that have been widely used in high-income countries, such as the Raven’s Progressive Matrices (Raven, 1986). The Raven’s test, which is a picture-based patterns test measuring fluid intelligence is thought to be relatively free of cultural biases without requiring language skills (Akresh et al., 2012, Borghans et al., 2008, Raven, 2000). However, some researchers believe there may still be inherent cultural biases within the Raven’s test as the pictures which are based on cultural practices may not be universal, sometimes even within the same country, let alone across all LMICs (Aslam et al., 2012, Benson, 2003, Owen, 1992).

In addition, few studies incorporated multiple standardized cognitive assessments to validate their study results. For example, one study included the Weschler Intelligence Scale to cross-validate their study results (Akresh et al., 2012), while another study utilized the Weschler Adult Intelligence Scale (Campos-Vazquez, 2018) to do the same. Other studies included and adapted the Peabody Picture Vocabulary Test and NEPSY block test (Fink et al., 2015), or used the Kaufman Assessment Battery for Children (Haile et al., 2016). Several studies utilized responses from subsets of questions included in large household surveys, usually measuring literacy or numeracy. Using literacy and numeracy scores as a proxy for cognitive ability has the potential for adding additional biases into the estimate, as gains in literacy and numeracy may be correlated with unobserved variables such as school quality. Greater efforts should be made to develop, test, and validate cognitive assessment tools in LMICs to ensure accurate and comparable measurements across countries and populations.

Our study has several limitations. First, as with any systematic review, ours is limited by the quality of included studies and any biases they may contain. Since there were few studies reporting on any one outcome measure of education or employment, results should be regarded with caution as generalizations may not be made without more robust evidence. While we systematically identified studies to be included in the *meta*-analysis, there were only a small number of studies found. With this number of studies we were not able to conduct a meta regression analysis and we could not rule out publication bias or reverse causality. Second, systematic reviews are inherently limited by their search strategies, databases searched, and the selected inclusion and exclusion criteria. We limited our systematic review to peer-reviewed literature published in English, eliminating gray literature such as economic working papers. We conducted a search across 8 databases to capture a wide range of literature across disciplines. Third, we limited our timeframe to studies conducted after 1999 to ensure current relevance of our findings. Therefore, we cannot assess economic trends in LMICs over time, such as changes in returns to cognitive ability since the 1980-1990′s when many LMICs were undergoing socio-political changes that impacted their economies. Fourth, it is not possible to determine causation between cognition, education, and socioeconomic status with the data presented here. However, these studies are still useful for explaining associations between cognition and economic outcomes or education. We were not able to analyze the impact of other confounding variables that may play a role in cognitive returns such as gender, as there weren’t sufficient numbers of studies examining this breakdown. Fifth, this analysis examined economic returns to cognition through individual wages and employment, and was not able to include other labor market outcomes such as family income. Further analysis should examine such outcomes that may be notable in LMICs, where a higher proportion of the workforce is seasonal or in the informal sector. There were not enough studies identified to examine the cognitive impact by age group. Thus, our results may present a simplified relationship of the overall impact of cognitive ability on schooling and employment outcomes. Despite these limitations, we believe we have identified and synthesized articles in a systematic and methodical manner to describe the educational and economic returns to cognitive ability across LMICs.

## 5 Conclusion

Greater cognitive ability is associated with greater and longer school enrollment and academic achievement in LMICs. Enhanced cognitive ability is also associated with increases in wages and greater employment opportunities in LMICs. In addition to supporting access to universal primary and secondary education, policies should focus on improving school quality so that learners acquire stronger cognitive skills to be better prepared for the labor market. Additionally, investments should be made for initiatives affecting *in utero* and early childhood development. These interventions, such as early infant nutrition programs and vaccinations, could have a high return on investment as they would boost early cognitive development and yield long-term returns to education and income for individuals as well as the broader economy in LMICs.

## CRediT authorship contribution statement

**Sachiko Ozawa:** Conceptualization, Methodology, Conceptualization, Methodology, Funding acquisition, Writing – original draft. **Sarah K. Laing:** Investigation, Writing – original draft. **Colleen R. Higgins:** Formal analysis, Investigation, Writing - review & editing. **Tatenda T. Yemeke:** Investigation, Writing - review & editing. **Christine C. Park:** Investigation. **Rebecca Carlson:** Investigation, Validation. **Young Eun Ko:** Investigation. **L. Beryl Guterman:** Methodology, Writing - review & editing. **Saad B. Omer:** Conceptualization, Methodology, Funding acquisition, Writing - review & editing.

## Declaration of Competing Interest

The authors declare that they have no known competing financial interests or personal relationships that could have appeared to influence the work reported in this paper.
